# Feasibility and prognostic value of stress-perfusion CMR in obesity

**DOI:** 10.1186/1532-429X-14-S1-O2

**Published:** 2012-02-01

**Authors:** Ravi Shah, Otavio R Coelho-Filho, Tomas G Neilan, Bobby Heydari, Ron Blankstein, Yucheng Chen, Eri Watanabe, Michael Jerosch-Herold, Raymond Kwong

**Affiliations:** 1 Brigham and Women's Hospital, Boston, MA, USA

## Background

Obesity is an emerging population at high risk for cardiac events for which robust stress imaging techniques are necessary. Stress-perfusion CMR has high image resolution and is less affected by tissue attenuation compared to nuclear scintigraphy. However, its prognostic value in obese patients has not been determined. We hypothesized that stress-perfusion CMR can effectively prognosticate obese patients with suspected myocardial ischemia.

## Methods

313 obese patients (body mass index BMI ≥ 30 kg/m2) referred for vasodilator stress-perfusion CMR for clinical indications between December 2001-August 2011 were evaluated. Stepwise Cox proportional hazards models were used to identify predictors of cardiac death or acute MI. Death was adjudicated as cardiac if there was antecedent MI or significant cardiac events within 30 days or if sudden and without a clear non-cardiac reason of death.

## Results

The median BMI was 35 (IQR 6.3) kg/m2, and average age was 56±12 years. Of 313 patients referred, 300 (96%) unOnly 13 (4%) failed to complete vasodilator stress-perfusion CMR (side effects to stress agent, 3; claustrophobia or patient request, 6; no IV access, 1; gating failure, 2; discovery of LV thrombus, 1). Thirty-two percent had abnormal stress perfusion and 34% had LGE. Mean LVEF was 57.2±14.6% and LV mass index 62.4±19.3 g/m2. Follow-up for vital status was available in all patients with a median follow-up of 3.61 years (range 0.5 to 7.9 years). There were 35 cardiac deaths and 34 acute MI. In a univariate Cox regression, patient age (hazard ratio HR = 1.08, P = .006), history of MI (HR = 9.84, P = .0001) or PCI (HR = 7.76, P = .0005), presence of ischemia (HR = 16.64, P = .0003), LGE (HR = 15.78, P = .01), and a presence of left bundle branch block (HR = 5.07, P = .02) predicted cardiac death or MI. In a stepwise multivariate Cox regression model, a history of percutaneous coronary intervention (LRχ2 14.92, P = .0001), presence (LRχ2 8.17, P = .004) and extent of Perf defect (LRχ2 21.5, P < .0001) were the only independent predictors of cardiovascular death or MI. A negative stress-perfusion CMR was associated with a 98% event-free survival during the study period.

## Conclusions

Assessment of obese patients with stress-perfusion CMR is effective and well-tolerated. The absence of a CMR stress-perfusion defect in obese patients portends an excellent negative cardiac event rate of 98%.

## Funding

RVS was supported by a Post-Doctoral Fellowship Grant from the American Heart Association (11POST000002). RYK and MJH are supported by grants from the National Institutes of Health (RO1HL091157 to RYK, 1RO1HL090634 to MJH).

**Figure 1:**
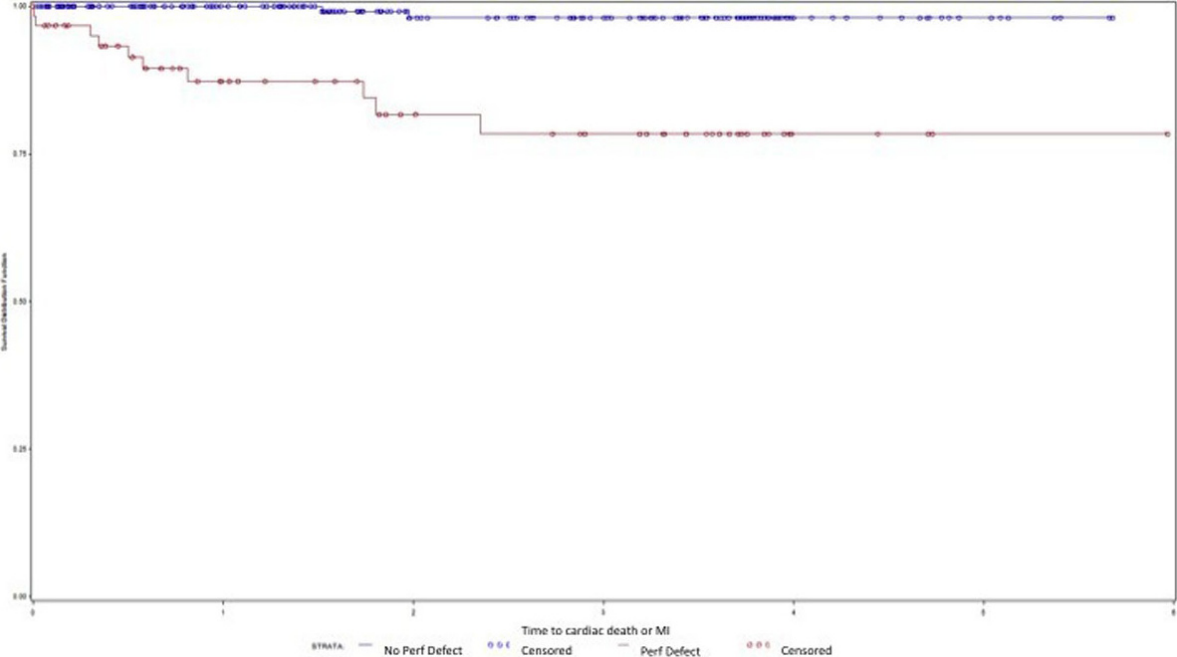
Event-free survival in obese patients stratified by perfusion defect.

